# Mechanometabolic Regulation in Cancer Progression: Lessons from Fibrotic Remodeling

**DOI:** 10.7150/ijbs.112325

**Published:** 2025-03-31

**Authors:** Chen-Yueh Wen, Po-Hung Chen, Renin Chang, Su-Boon Yong, Chin-Yuan Yii, Yen Chin, Chia-Jung Li

**Affiliations:** 1 Division of Urology, Show Chwan Memorial Hospital, Changhua 500, Taiwan.; 2 Department of Emergency Medicine, Kaohsiung Municipal Min-Sheng Hospital, Kaohsiung 802, Taiwan.; 3 Department of Emergency Medicine, Kaohsiung Veterans General Hospital, Kaohsiung, Taiwan.; 4 Department of Allergy and Immunology, China Medical University Children's Hospital, Taichung 404, Taiwan.; 5 Research Center for Allergy, Immunology, and Microbiome (A.I.M.), China Medical University Hospital, Taichung 404, Taiwan.; 6 Department of Internal Medicine, Landseed International Hospital, Taoyuan, Taiwan.; 7 Department of Internal Medicine, Kaohsiung Municipal United Hospital, Kaohsiung, Taiwan.; 8 Department of Obstetrics and Gynaecology, Kaohsiung Veterans General Hospital, Kaohsiung 813, Taiwan.; 9 Institute of Biopharmaceutical Sciences, National Sun Yat-sen University, Kaohsiung 804, Taiwan.

SLC2A3 (GLUT3) is a high-affinity glucose transporter that plays a crucial role in cellular energy metabolism. It is particularly important in metabolically active tissues, where it ensures sufficient glucose uptake even under low-glucose conditions 1. Unlike other glucose transporters, SLC2A3 has a higher affinity for glucose, enabling cells to maintain energy homeostasis in challenging metabolic environments 2. Emerging research has revealed that SLC2A3 is frequently upregulated in cancer cells, allowing them to meet increased energy demands necessary for rapid proliferation and survival 3. Furthermore, SLC2A3 is implicated in hypoxia adaptation and oxidative stress responses, further highlighting its significance in tumor progression 4. Due to these characteristics, SLC2A3 has garnered attention as a potential therapeutic target in various malignancies, including gastric cancer. A recent study titled “TAGLN-RhoA/ROCK2-SLC2A3-mediated Mechano-metabolic Axis Promotes Skin Fibrosis” explored the role of the mechanotransduction and metabolic pathways in skin fibrosis. The authors identified the TAGLN-RhoA/ROCK2-SLC2A3 axis as a pivotal factor in the pathogenesis of skin fibrosis. This axis, upregulated in fibrotic tissues, enhances cellular contraction and metabolic reprogramming, leading to fibroblast proliferation, migration, and extracellular matrix deposition. By modulating key proteins such as TAGLN, RhoA, ROCK2, and SLC2A3, the axis contributes to the progression of skin fibrosis 5.

Building on this, our study investigates SLC2A3's role in gastric cancer (STAD), using multi-omics approaches (TCGA, single-cell RNA sequencing, spatial transcriptomics) to reveal its association with tumor progression, fibrosis, immune modulation, and drug response prediction—offering novel insights into mechanometabolic regulation in cancer. First, we observed a significant upregulation of SLC2A3 expression in gastric cancer tissues, particularly in patients with advanced disease. This increased expression correlated strongly with fibrosis and tumor progression, suggesting a potential role in promoting an aggressive tumor phenotype. To better understand the molecular landscape associated with SLC2A3, we conducted an extensive analysis of SLC2A3 expression in STAD patients using data from The Cancer Genome Atlas (TCGA). We visualized the top 20 most affected genes through a waterfall plot, providing insights into the broader genetic alterations associated with STAD. Genetic variation analysis revealed a strong association between SLC2A3 expression and frequently mutated genes in STAD, including TTN and TP53, both of which are known to drive cancer progression and genomic instability (Fig. 1A). To gain further insights into the expression patterns of SLC2A3, we analyzed its levels across different sample types, cancer stages, and metastatic stages using TCGA data (Fig. 1B-D). Our findings revealed that SLC2A3 expression progressively increased as the disease advanced, with the highest expression levels observed in metastatic cases. To assess the prognostic significance of SLC2A3, we performed Kaplan-Meier survival analysis and log-rank tests. These analyses demonstrated that high SLC2A3 expression was significantly associated with poorer survival outcomes in STAD patients, reinforcing its potential as a prognostic biomarker (Fig. 1E). We further validated SLC2A3 expression in tumor and non-tumor tissues using spatial transcriptomics (ST) data from gastric cancer patients. By integrating hematoxylin and eosin (H&E) staining with cellular marker annotation, we were able to identify tumor-specific regions with high precision. Unsupervised clustering analysis revealed that SLC2A3 expression closely mirrored the distribution of known cancer stem cell markers such as SOX2 and GABRP (Fig. 1F). Quantitative analysis confirmed that SLC2A3 levels were significantly elevated in tumor regions compared to non-tumor areas, suggesting a role in maintaining cancer stemness and tumor growth (Fig. 1G).

To further characterize SLC2A3 expression at the single-cell level, we analyzed a publicly available single-cell RNA sequencing dataset (GSE167297), which included tumor cells and various immune cell populations. Our results showed that SLC2A3 was highly enriched in macrophages, a key component of the tumor microenvironment (Fig. 1H). Gene set enrichment analysis (GSEA) demonstrated a strong correlation between SLC2A3 expression and inflammatory response pathways, particularly IL2/STAT5 signaling (Fig. 1I). Additionally, GSEA indicated a positive association between high SLC2A3 expression and macrophage activation, further supporting its role in shaping the tumor microenvironment (Fig. 1J). Functional analysis revealed that elevated SLC2A3 levels were linked to increased lymphocyte infiltration and macrophage regulation, as well as a heightened TGFb response. However, no significant differences were observed in TIL regulation, Th17 response, or IFN-γ signaling, suggesting that SLC2A3's influence on the immune microenvironment is primarily through macrophage-driven pathways (Fig. 1K). To further explore this relationship, we analyzed the correlation between SLC2A3 expression and macrophage subtypes, specifically M0, M1, and M2 macrophages. While M0 and M1 macrophages exhibited a positive correlation with SLC2A3, M2 macrophages showed no significant association, indicating a potential selective role in pro-inflammatory and tumor-promoting macrophage populations (Fig. 1L). In addition to its role in tumor progression and immune modulation, we explored the potential clinical applications of SLC2A3 as a therapeutic target. Drug evaluation using data from the CTRP Pharmacogenomic Database identified ten drugs for which high SLC2A3 expression predicted increased sensitivity and ten drugs for which high expression predicted resistance (Fig. 1M). This analysis, validated through a 10-fold cross-validation approach, demonstrated strong predictive accuracy for drug response data in cancer cell lines. These findings underscore the potential of leveraging SLC2A3 expression profiles to guide personalized therapeutic strategies for STAD patients.

The insights gained from studying SLC2A3 in gastric cancer provide a valuable framework for understanding its role in STAD. The shared molecular mechanisms highlight the potential of targeting SLC2A3 as a therapeutic strategy in both conditions. By elucidating the function of SLC2A3 in STAD, our research aims to develop targeted therapies that could improve clinical outcomes for patients with this aggressive cancer. Notably, previous studies have reported that SLC2A3 is not only upregulated in tumor-associated macrophages but also exhibits elevated expression in other immune cells, including neutrophils and T cells, particularly under hypoxic conditions 6, 7. This suggests that SLC2A3 may contribute to broader immune cell reprogramming within the tumor microenvironment, potentially promoting an immunosuppressive state that favors tumor progression.

While our findings highlight the TAGLN-RhoA/ROCK2 axis as a regulator of SLC2A3 expression, direct evidence in gastric cancer remains limited. Fibrosis-related pathways, such as TGF-β signaling, extracellular matrix (ECM) remodeling, and YAP/TAZ activation, may also modulate SLC2A3 in STAD, given their established roles in tumor stiffness and progression. Future studies should validate this axis in gastric cancer models, potentially through ROCK2 inhibitors or SLC2A3 knockdown experiments, to confirm its mechanistic relevance. Beyond macrophages, SLC2A3's upregulation in T cells and neutrophils under hypoxic conditions suggests a broader immunomodulatory role (6,7). This may contribute to an immunosuppressive tumor microenvironment, warranting further investigation into its effects on anti-tumor immunity. Therapeutically, SLC2A3 inhibitors could synergize with chemotherapy (e.g., enhancing gemcitabine sensitivity) or immunotherapy (e.g., reversing macrophage-driven immunosuppression), offering a combinatorial approach to improve STAD outcomes. Given these findings, future studies should explore the precise role of SLC2A3 across different immune cell populations and investigate whether its modulation could enhance anti-tumor immunity. These insights will be critical for developing novel immunotherapeutic approaches targeting SLC2A3 in gastric cancer and beyond.

## Figures and Tables

**Figure 1:**
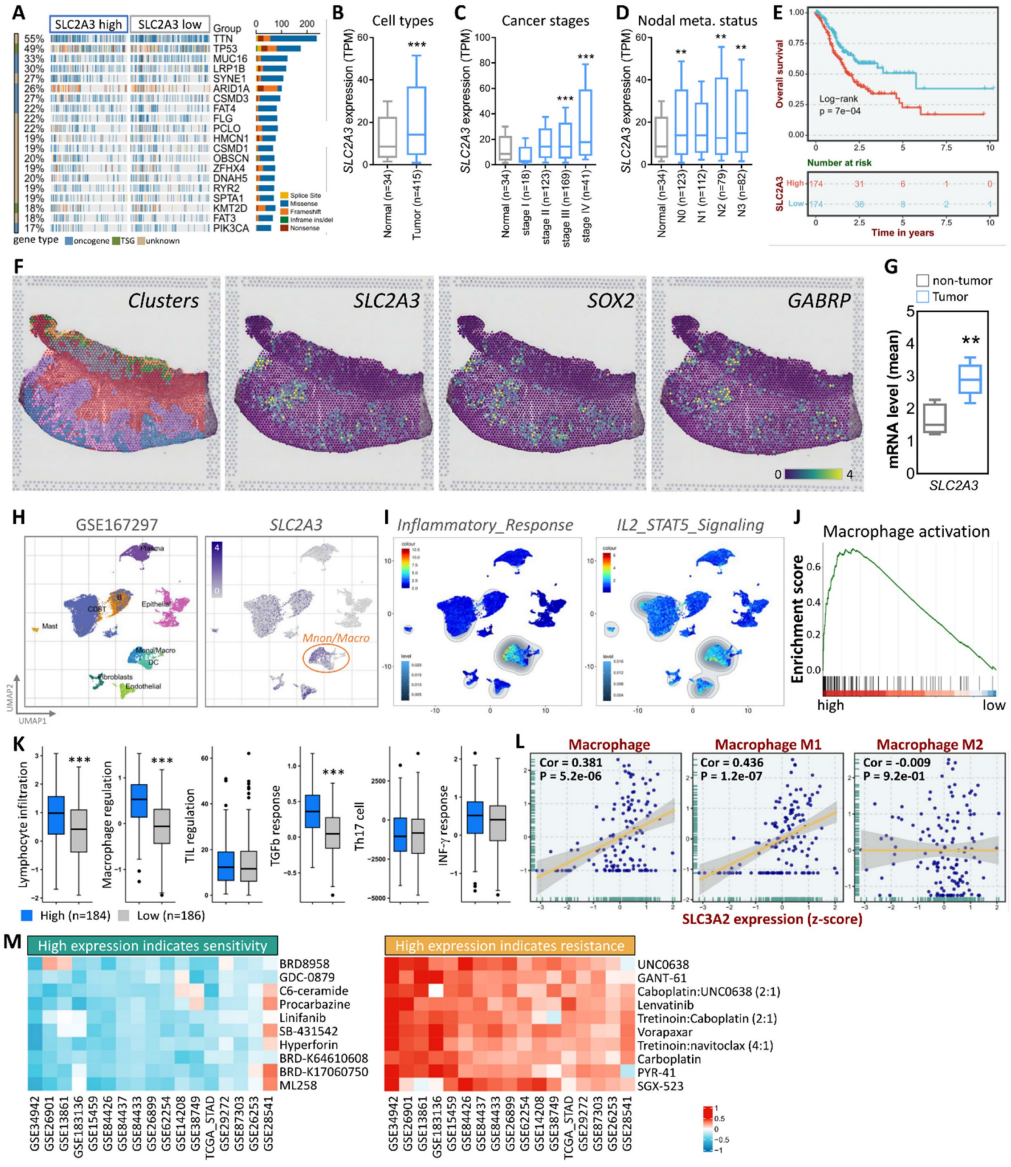
** SLC2A3 expression and its clinical significance in gastric cancer.** (A) Waterfall plot showing the top 20 most affected genes in STAD patients (n=375) based on TCGA data. (B-D) SLC2A3 expression levels across sample types (tumor vs. normal, n=375 vs. 32), cancer stages (I-IV, n=375), and metastatic stages (M0 vs. M1, n=350 vs. 25), analyzed via Wilcoxon rank-sum tests (*p<0.05, **p<0.01). (E) Kaplan-Meier survival analysis of SLC2A3 expression in STAD patients (n=375), with orange curve representing high expression and blue curve low expression (median cutoff), assessed by log-rank test (p<0.01). (F) Spatial transcriptomics analysis with H&E staining and cellular marker annotation identifying tumor-specific regions (n=5 patients). (G) Unsupervised clustering analysis showing SLC2A3 colocalization with cancer stem cell markers (SOX2, GABRP). (H) Single-cell RNA sequencing (scRNA-seq) analysis of SLC2A3 expression in gastric cancer samples (GSE167297, n=10). (I-J) Gene set enrichment analysis (GSEA) of SLC2A3 expression in inflammatory response (IL2/STAT5 signaling) and macrophage activation (n=375, FDR<0.05). (K) Functional analysis of SLC2A3-related immune pathways (n=375). (L) Correlation between SLC2A3 expression and macrophage subtypes (M0, M1, M2; n=375, Pearson correlation, *p<0.05). (M) Drug response analysis using the CTRP Pharmacogenomic Database (n=500 cell lines), with sensitivity (AUC<0.5) and resistance (AUC>0.7) determined via 10-fold cross-validation (95% CI reported).
